# Public Health Risks of PFAS-Related Immunotoxicity Are Real

**DOI:** 10.1007/s40572-024-00441-y

**Published:** 2024-03-25

**Authors:** Abigail P. Bline, Jamie C. DeWitt, Carol F. Kwiatkowski, Katherine E. Pelch, Anna Reade, Julia R. Varshavsky

**Affiliations:** 1 Social Science Environmental Health Research Institute, Northeastern University, Boston, MA 02115 USA; 2 Silent Spring Institute, Newton, MA 02460 USA; 3 Department of Environmental & Molecular Toxicology, Oregon State University, Corvallis, OR 97331 USA; 4 Department of Biological Sciences, North Carolina State University, Raleigh, NC 27695 USA; 5 Natural Resources Defense Council, San Francisco, CA 94104 USA; 6 Departments of Health Sciences and Civil and Environmental Engineering, Northeastern University, Boston, MA 02115 USA

**Keywords:** Immunosuppression, Vaccine, Per- and polyfluoroalkyl substances, Risk assessment, Clinical guidance, Population health

## Abstract

**Purpose of Review:**

The discovery of per- and polyfluoroalkyl substances (PFAS) in the environment and humans worldwide has ignited scientific research, government inquiry, and public concern over numerous adverse health effects associated with PFAS exposure. In this review, we discuss the use of PFAS immunotoxicity data in regulatory and clinical decision-making contexts and question whether recent efforts adequately account for PFAS immunotoxicity in public health decision-making.

**Recent Findings:**

Government and academic reviews confirm the strongest human evidence for PFAS immunotoxicity is reduced antibody production in response to vaccinations, particularly for tetanus and diphtheria. However, recent events, such as the economic analysis supporting the proposed national primary drinking water regulations and clinical monitoring recommendations, indicate a failure to adequately incorporate these data into regulatory and clinical decisions.

**Summary:**

To be more protective of public health, we recommend using all relevant immunotoxicity data to inform current and future PFAS-related chemical risk assessment and regulation. Biological measures of immune system effects, such as reduced antibody levels in response to vaccination, should be used as valid and informative markers of health outcomes and risks associated with PFAS exposure. Routine toxicity testing should be expanded to include immunotoxicity evaluations in adult and developing organisms. In addition, clinical recommendations for PFAS-exposed individuals and communities should be revisited and strengthened to provide guidance on incorporating immune system monitoring and other actions that can be taken to protect against adverse health outcomes.

**Supplementary Information:**

The online version contains supplementary material available at 10.1007/s40572-024-00441-y.

## Introduction

Concern over the health and environmental effects of the large class of human-made chemicals known as PFAS (per- and polyfluoroalkyl substances) is growing rapidly [1]. PFAS have been detected worldwide in air, water, soil, and indoor environments as a result of the production, use, and disposal of PFAS-containing products. Such products include building materials, household products, textiles, electronics, personal care products, industrial processing aids, and more [2]. PFAS are used in these products for the myriad technical functions they provide, for example, acting as water and oil repellants, surfactants, emulsifiers, and friction reducers. However, scientific research has revealed a long list of confirmed and suspected adverse health effects in humans, laboratory models, and wildlife as a result of exposure to PFAS [3, 4••, 5]. These include several cancers (e.g., of the kidney and testes), decreased fertility, hormone disruption, liver disease, immune system dysfunction, and more—most of which have been detected through study of only a few well-known PFAS (e.g., perfluorooctanoic acid (PFOA) and perfluorooctane sulfonic acid (PFOS)). Nearly all of the remaining thousands of PFAS have been studied minimally or not at all [6–8].

In developing health-based guidelines for PFAS, several US federal and state agencies, as well as the European Food Safety Authority, have recognized immunotoxicity as one of the most sensitive outcomes of PFAS exposure, meaning that adverse effects are seen at lower doses than for other outcomes [9•, 10•, 11–17]. Recent academic and governmental reviews concluded that PFAS exposure is linked to immune system suppression [18, 19, 20•, 21•, 22, 23]. Reduced antigen-specific antibody responses (e.g., in response to vaccinations) provided the strongest evidence for immunotoxicity from both human and experimental animal studies [22, 23].

Despite these findings, some have doubted the validity and utility of immunotoxicity endpoints in informing risk management decisions. In a regulatory context, the US Environmental Protection Agency (EPA) recently concluded it could not quantify immune system effects in its economic analysis for establishing PFAS maximum contaminant levels (MCLs) [24]. This contributed to an underestimate of the economic benefits of regulation, as acknowledged by the agency [24]. Similarly, a 2022 clinical guidance document for PFAS-impacted populations generated from a committee appointed by the National Academies of Science, Engineering, and Medicine (NASEM) provided minimal recommendations related to the immune system, essentially dismissing community concerns [4••]. In both cases, the rationale for not fully addressing PFAS immunotoxicity was that the observed biological markers of effect did not have clear implications for clinical disease risk and therefore could not be relied upon for decision-making. In this paper, we expand on the context surrounding these decisions to not appropriately include immunotoxicity outcomes and describe their public health consequences. We argue that the available immunotoxicity data on PFAS are actionable and should be used by decision-makers in both regulatory and clinical settings.

## Adverse Effects of PFAS on the Immune System—State of the Science

The health of an individual is dependent on immune system homeostasis. When in balance, the immune system can detect a threat to the host, mount a response, and once the threat is resolved, repair any tissue damage and return to a resting state [25]. The immune system is dispersed throughout, and integrated within, most tissue types and organ systems. As such, it can be readily targeted by toxicants through nearly any exposure route. Since the immune system is composed of a diverse range of cell types with various functions, toxicant exposures can lead to immune dysfunction in myriad ways, ultimately leading to immunosuppression, inappropriate immune activation, or both [26]. It is also well understood that when the immune system is perturbed during development, such as from exposure to immunotoxicants, effects are likely more severe and more long-lasting than from perturbations that occur during adulthood [27]. Mounting evidence points to PFAS as potent human immunotoxicants.

The strongest epidemiological evidence for PFAS-associated immunotoxicity is reduced antibody production in response to vaccinations, particularly in children receiving tetanus and diphtheria vaccines [20•, 21•, 22]. Antibodies are a key component of the adaptive immune system involved in responding to and limiting damage from infectious agents and toxicants [28]. Experimental animal studies also support decreased antigen-specific antibody responses caused by exposure to certain PFAS [22]. Additionally, PFAS exposure has been associated with an increased risk of respiratory tract and gastrointestinal infections in experimental animal and human studies, particularly in children with in utero maternal PFAS exposures [18, 20•]. These reviews and more recent studies highlight the immunosuppressive effects of PFAS [29, 30]. PFAS exposure may also contribute to inappropriate immune activation, though the evidence base is less consistent [20•, 22]. In particular, PFAS exposure may worsen pre-existing asthma and allergic reactions in the lungs [20•].

Furthermore, exposure to PFAS (and other exogenous chemicals) is known to exert a wide range of adverse effects on many other systems and processes of the body, some of which may be linked to disrupted immune homeostasis [31]. We highlight here a few examples demonstrating that PFAS-induced immune system changes may be initiating or contributing events in disease processes beyond the immune system. PFAS have been shown to be immunosuppressive to trophoblast cells, suppressing production of inflammatory proteins necessary for establishing proper blood flow between the placenta and maternal endometrium [32]. This immune-mediated mechanism in the placenta could underlie the observed association between PFAS exposure and increased risk of preeclampsia in humans [33]. PFAS can also activate Kupffer cells, the tissue-resident macrophages in the liver, which can lead to the release of inflammatory cytokines and cell proliferation that promotes liver cancer [34]. Such immune cell-mediated effects may also contribute to PFAS liver toxicity consistently observed in human and experimental animal studies [34, 35]. In a study of pre- and postnatal exposure to PFAS, a positive relationship between prenatal PFOA levels and IL-1beta, a pro-inflammatory cytokine was observed [36]. Levels of the IL-1beta also were linked with a larger waist circumference in the exposed children, suggesting that increased inflammation contributed to an unfavorable metabolic profile [36].

## PFAS Immunotoxicity and Decision-Making

Given the importance of proper immune system functioning, it is necessary to effectively use and act on available immunotoxicity data to protect individual and public health. Chemical risk assessment frameworks for non-cancer endpoints typically follow discrete steps, including identifying the most sensitive endpoints (hazard identification), determining their dose responsiveness, and applying uncertainty factors (e.g., to account for limitations in the existing literature database). These steps determine a level of daily exposure below which no adverse effects are anticipated (typically called a reference dose). For example, EPA typically determines an oral reference dose (RfD), which is defined as the daily oral exposure to the human population that is likely to be without appreciable lifetime risk of deleterious effects. The RfD is then used in risk management decisions, such as setting drinking water MCLs, the highest amount of a chemical that is legally allowed. Here, we examine how immunotoxicity data are generated and used in health-based hazard identification, risk assessment, and clinical decision-making contexts, and the associated consequences of neglecting to account for sensitive immune endpoints.

### Lack of Immunotoxicity Testing Requirements and Failure to Use Available Data Hinder Hazard Identification and Risk Assessment

Other than skin sensitization, functional immunotoxicity testing is not routinely performed on new chemicals in the USA or Europe [20•, 37]. Instead, toxicity tests generally focus on gross organ damage and lethality occurring at high doses that are typically unrepresentative of human exposure scenarios [38, 39]. While these tests can identify some effects on major immune system organs, like the spleen and thymus, they do not rule out effects on immune system function, as is assumed by regulatory frameworks. For example, the ability of the immune system to function properly in response to an immune challenge (like a bacterial infection) can still be impacted even if immune organs do not show obvious changes [26, 40]. Recognizing the limits of routine toxicity tests and the importance of assessing functional immune endpoints, the EPA established a test guideline for evaluating suppression of the immune system using a T-cell-dependent antibody response (TDAR) assay in 1998 [41]. The EPA briefly required that all new pesticides be tested for immunosuppressive effects using this test guideline, which can readily be incorporated into other subchronic or chronic toxicity tests. However, the pesticide industry argued that it was unnecessary, at which point the requirement was dropped [42, 43]. Testing for immunosuppression has never been a requirement for industrial chemicals [44]. EPA did identify immunotoxicity as a relevant endpoint in the 2021 National PFAS Testing Strategy document, which outlines plans to require companies to perform toxicity testing on current-use PFAS under the Toxic Substances Control Act (TSCA) [45]. However, EPA did not require immunotoxicity testing in the two testing orders issued so far [46].

Routine testing has also failed to account for developmental immunotoxicity, which is equally important, given that immunotoxic effects may be particularly evident if exposure occurs during a sensitive window of development and/or when an individual is faced with additional immune challenges [26]. Evaluation of developmental immunotoxicity is not required by EPA or other regulatory bodies. The result of lax functional and developmental immunotoxicity testing requirements is that many substances, including PFAS, are allowed in the marketplace with relatively little information known about their true immune hazard potential. Thus, it was decades after they came into use and widespread human exposure had occurred that immunotoxic effects of PFAS were publicly identified through independent research.

While these independent research efforts have clearly established PFOA and PFOS as immunotoxicants, the continued lack of regulatory immunotoxicity testing requirements has resulted in a dearth of robust immunotoxicity data for other PFAS to which the population is exposed. For example, ≥ 99% of Americans have detectable levels of perfluorohexane sulfonic acid (PFHxS) in their blood [47]. Yet the EPA recently noted that of 20 experimental animal studies available for PFHxS, there were none that evaluated immune system function [48]. This is surprising given the similarity of PFHxS to PFOS and the availability of epidemiological data linking PFHxS exposure to immunosuppression and specifically, decreased antibody response to vaccination [48]. In situations like this, where there is an absence of robust immunotoxicity data, evidence from well-studied PFAS could be used to make inferences about structurally similar PFAS, an approach sometimes referred to as “read across.” While a read-across approach is allowed for use in risk assessment under TSCA, it has been more frequently utilized by industry to avoid toxicity testing rather than as a means for EPA to restrict likely hazardous chemicals [49, 50].

Uncertainty factors are often used in risk assessment to avoid underestimating risk when insufficient data or knowledge are available. When extrapolating from a PFAS with available immunotoxicity data to another structurally similar PFAS lacking these data, the addition of an uncertainty factor acknowledges the common effects of structurally related PFAS while also providing for unknown differences in the magnitude of effects. Risk assessors have occasionally, but not always, added an uncertainty factor to account for the lack of available immunotoxicity data for some PFAS, including PFHxS (see Supplemental Table 1 for a summary of select state and federal risk assessments that have been conducted for individual PFAS). If an uncertainty factor is not used when extrapolating PFAS immunotoxicity data to untested PFAS, the resulting RfD and MCL may be an order of magnitude higher and the health risk of the untested PFAS would likely be underestimated.

### Omission of Immunotoxic Effects Undermines Risk Management Decisions

Efforts have been made to protect against PFAS immunotoxicity in some chemical management and decision-making contexts. Protective actions have included several US state agencies choosing immunosuppression endpoints as the critical effect for calculating reference doses for PFOA and PFOS. For example, MI and NJ used the suppression of a plaque-forming cell response in mice, and MN, NH, and WA used the suppression of IgM response in mice as the basis for the PFOS reference dose derived by each agency [13–15, 17, 51] (Supplemental Table 1). More recently, the EPA used a suppressed antibody response to vaccines observed in human epidemiological studies as the basis for the interim lifetime health advisories for PFOA and PFOS [52••, 53••].

Despite these efforts, the use of immunotoxicity endpoints, particularly reduced vaccine response, as the basis for setting PFOA and PFOS interim health advisories, has drawn several new critiques [19, 54]. In particular, in a review funded by 3 M, Antoniou et al. suggested that reductions in vaccine-induced antibody levels without a concurrent rise in infection rates should not warrant regulatory action [54]. This argument runs counter to how experts have defined immunosuppression as “a reduced ability of the immune system to respond to a challenge from a level considered normal, regardless of whether clinical disease results” [55]. It is also unsupported by federal regulations, which define immunotoxicity under the TSCA § 799.9780 as “…the ability of a test substance to suppress immune responses that *could enhance the risk* of infectious or neoplastic disease, or to induce inappropriate stimulation of the immune system, thus *contributing* to allergic or autoimmune disease [*emphasis added*]” [56]. Moreover, the discrediting of PFAS-associated antigen-specific antibody response data is also contradictory to how similar data are used in pharmaceutical development, both for assessing potential immunotoxicity as well as for testing efficacy of immune-modulating drugs [42, 57, 58]. It is illogical for antibody response assays to be deemed reliable when assessing immune system effects of pharmaceuticals, yet questionable when applied to assessing immune effects of environmental chemicals known to be present in humans at bioactive concentrations.

The dismissal of effects on antibody responses to vaccines as actionable also appeared in EPA’s economic analysis supporting the proposed national primary drinking water regulations for six PFAS. In the proposal, the EPA acknowledged that PFAS cause immunotoxic effects but argued that these “biomarker” responses (i.e., reduced vaccine-induced antibody titers) could not be considered in the economic analysis due to the lack of clear impact on public health [59]. Thus, the economic benefit (health care cost savings) to people who would be protected from further contamination was underestimated, putting the proposed regulations at risk of being weakened or denied. In this case, the EPA labeled the reduced vaccine response as a “health effect” rather than a “health outcome,” a distinction the agency used in the economic analysis without explanation [24]. The use of labels to distinguish between types of health data (subclinical vs clinical, mechanistic vs apical, biomarker vs disease, health effect vs health outcome) is common, but not well defined and frequently not justified. Yet, such labels are often used to determine if an observed health effect is actionable, such was the case in the economic analysis where this “biomarker” of effect was determined to not be quantifiable.

In contrast, the EPA has historically taken action on other “subclinical” health effects in economic analyses. For example, when evaluating the costs and benefits of the Clean Air Act Amendments in 1990, the EPA noted a positive association between blood lead levels and blood pressure and separately, that increases in blood pressure were linked to increased risk of a first-time cardiovascular disease event, stroke, or mortality [60]. In this case, the EPA calculated the benefits of reduced first-time cardiovascular disease events or stroke based on lead-related effects on blood pressure. Similarly, in the PFAS economic analysis, the EPA quantified the benefits of protecting against several other PFOA and PFOS-related health endpoints, including lipid level changes [24]. Specifically, the EPA recognized total cholesterol (another biomarker) as a predictor of cardiovascular disease and quantified the economic benefit of avoiding additional cases of cardiovascular disease events [24]. It is unclear why the EPA did not apply the same reasoning to PFAS-associated decreases in vaccine-induced antibody responses, which is an accepted indicator of immunosuppression. This decision is particularly concerning, given the agency has acknowledged that PFAS exposure is associated with “reduced ability of the body’s immune system to fight infections, including reduced vaccine response,” as well as some corroborating evidence that PFAS exposure is associated with increased susceptibility to common infections [20•].

We argue that to require data that directly link PFAS exposure to reduced vaccine-induced antibody response and to increased rates of those vaccine-controlled diseases is unreasonable, given the widespread adoption of vaccines and other public health measures that keep these diseases at a minimum. Perhaps more importantly, waiting to regulate PFAS until we have clear evidence of higher population-level rates of any type of infection, much less those with vaccines available, puts the entire population at risk and is contrary to protecting public health.

### Consequences of Discounting Immunotoxicity in a Clinical Setting

Even though the EPA and others have acknowledged that PFAS can reduce the ability of the immune system to fight infection, this risk has not been used as an actionable endpoint in clinical guidance documents. In 2021, in response to community requests for improved clinician guidance on PFAS exposure and response, a NASEM ad hoc committee was tasked with examining health outcomes associated with the most widely studied PFAS and to “make recommendations to the CDC on who, when, how, and what to test, as well as the risks of testing” [61].

Upon reviewing the scientific literature, the committee categorized health outcomes associated with PFAS exposure according to the amount of evidence for each. With regard to immune-related outcomes, the committee determined there was “sufficient evidence for an association of PFAS exposure with decreased antibody response to vaccination or infection, and limited suggestive evidence of an association with ulcerative colitis” [4••]. Despite these conclusions, the only clinical care recommendations for immunotoxicity endpoints were to screen those in the highest exposure category (> 20 ng/mL in plasma or serum) at well-visits for ulcerative colitis, an immune endpoint the committee concluded had limited evidence. Notably, NASEM’s lack of guidance regarding vaccine-induced antibody responses fails to acknowledge, and is not in alignment with, earlier recommendations from the National Research Council (NRC) [62]. The NRC had previously proposed that all people exposed to an immunotoxicant should be checked by a physician once to twice per year for several immune-related endpoints (see Supplemental Table 2) [62], including secondary antibody responses to tetanus and diphtheria toxoid antigens. Also of note is that the NASEM report recommended lipid panel screenings due to increased risk of dyslipidemia from PFAS exposure, an endpoint that had the same level of evidence as decreased antibody responses and could also be considered as a “subclinical” or “biomarker” of effect.

The lack of clinical recommendations for immunotoxic endpoints in NASEM’s 2022 guidance document not only inexplicably ignores the 1992 NRC recommendations, but also ignores concerns expressed by PFAS-impacted community members. Several residents of communities impacted by high levels of PFAS shared important comments about the need to address adverse effects of PFAS on the immune system during the NASEM Committee’s Town Halls [4••]. One community member shared how her neighbor’s pediatrician monitored the vaccine response of their highly exposed child. When the child did not mount an effective response to the vaccine, an additional booster, not normally required, was offered. Several other community members suggested that PFAS exposure may have made them more vulnerable to developing COVID-19 and that information was important for designing COVID-19 interventions and public health protocols for their communities. Notably, PFHxS was recently associated with reduced antibody levels in response to SARS-CoV-2 infection in pregnant women [63].

NASEM’s recommendations are also inconsistent with clinical guidance provided for other agents known to be immunosuppressive, such as certain pharmaceuticals. Similar to PFAS, pharmaceuticals like cyclosporine decrease antibody response, but do not generally cause clinical symptoms or disease of the immune system [64, 65]. However, their long-term use can increase the risk for cancer [66, 67] and infectious disease [68], risks also associated with chronic PFAS exposure [22, 69]. Due to the risks for people taking immunosuppressive pharmaceuticals, clinicians are provided with warnings that vaccines may be less effective and infections may be more common in these patients [70]. The acceptance of the clinical implications of intentional immunosuppression for pharmaceuticals but rejection of the importance of unintentional immunosuppression by industrial chemicals reflects broader inconsistencies regarding the treatment of immunosuppression in different decision contexts.

## Public Health Implications: Small Changes Have Big Impacts

The health of a population is dependent on healthy immune systems in individuals. Changes in indicators of disease, whether they are biological markers of clinical disease status or other measures of dysfunction, can have significant public health implications. Small changes at the individual level can have large societal and economic impacts at the population level. For example, developmental exposure to polybrominated diphenyl ethers (PBDEs) has been shown to reduce IQ levels. While several IQ points may not seem significant in individuals, shifting the entire population (or sensitive subpopulations) closer to disease status (i.e., learning disabilities) has a significant public health and financial burden on society [71, 72]. Population-level shifts in IQ have been used in cost burden analyses, with PBDE-related IQ changes cited as a leading driver of the total US disease cost associated with endocrine-disrupting chemicals [72]. Other examples exist. Exposure to metabolism-disrupting chemicals, in combination with the normal increase in insulin resistance that occurs during pregnancy, can put more pregnant people over clinically defined thresholds of disease status with respect to gestational diabetes [73]. Environmental chemicals that elevate blood pressure, or that decrease glomerular filtration rate, can also move susceptible populations closer to physiologically defined thresholds of clinical disease outcomes, such as hypertension and chronic kidney disease, respectively [74, 75].

Similarly, small shifts in immune function in individuals (e.g., decreased antibody levels) can have major impacts at the population level, such as increased risk of infection from community-based pathogens and other immune-related diseases [26]. In contrast to non-communicable diseases like hypertension or kidney disease, where the risk is primarily experienced at the individual level, population-level shifts in immune function can be compounded by the communicable nature of infectious diseases. Increased susceptibility in individuals to communicable infections associated with PFAS exposure, such as SARS-CoV-2, influenza, and other pathogens, can affect disease risk in the rest of the population, indicating that disruptions to immune system endpoints pose a unique and urgent public health risk. An additional concern with PFAS-associated immunosuppression is that a shift in the number of people that can mount a sufficient immune response to a vaccine will affect the number of people needing a vaccine to reach community immunity [76, 77].

One way to understand societal risks of immune dysfunction is to examine social and economic impacts of infectious diseases in the general population [25]. In 2019, the age-adjusted death rate for influenza and pneumonia was 12.3%, which was the ninth leading cause of death in the USA for that year [78]. In 2013, the last period for which data were aggregated, it was estimated that nearly $20 billion was spent on pneumonia and influenza health care [79]. Along with these statistics are accumulating data indicating that exposure to contaminants that target the immune system, especially when it is developing, increases risks of myriad chronic diseases [31]. This is particularly true for sensitive subgroups, such as children and the elderly. Aging has long been associated with decreased immune function (i.e., immunosenescence) and adverse clinical outcomes, particularly increased viral and bacterial infections [61, 75]. Several studies have reported that responses to vaccinations are lower in the elderly compared to younger adults [80, 81]. Monitoring and acting on changes in immune biomarkers is a useful strategy to protect public health, especially in these vulnerable populations [82]. In addition, protecting the immune system can potentially protect against several other diseases, given that “upstream” immune system changes (e.g., inflammation) can lead to multiple downstream clinical outcomes, including metabolic outcomes (e.g., diabetes, fatty liver disease, and heart disease), pregnancy complications (e.g., preterm birth and preeclampsia), and cancer [31].

## Recommendations

This commentary highlights several instances where PFAS-associated immunotoxicity data were discounted or not fully incorporated into various decision-making contexts and the ensuing consequences that may arise from those decisions. Specifically, we identified that there are insufficient immunotoxicity testing requirements, and there has been an inconsistent accounting for immunotoxicity in regulatory and clinical decision-making contexts. These deficits put public health at risk.

Moving forward, it is essential that informative immunotoxicity data for industrial chemicals like PFAS continue to be generated and that the entirety of the body of evidence be used and acted upon to protect public health. Importantly, biochemical measures of immune system effects should be treated as valid and informative markers of health outcomes and risk, consistent with how they are treated for other organ systems (e.g., cardiovascular) and in other regulatory contexts (e.g., pharmaceuticals). Furthermore, the key characteristics framework recently developed for immunotoxicants—which outlines the properties of chemicals that confer potential immunotoxicity—is useful for the identification, organization, and integration of mechanistic data into these review processes [83, 84].

While most PFAS lack immunotoxicity testing data, it is particularly astounding that there are so few functional immune studies available for highly studied and frequently detected PFAS like PFHxS and perfluorodecanoic acid (PFDA). To help fill the current data gap, toxicity testing requirements should be designed to examine immune system effects more thoroughly, including developmental immunotoxicity. For example, the EPA should require functional immunotoxicity testing for all new chemicals, as it previously did for pesticides, instead of allowing for the assumption of safety in the absence of data. EPA should also accelerate the pace of issuing testing orders for current-use PFAS under the National PFAS Testing Strategy and ensure that functional immunotoxicity tests are included in future orders. Given the evidence of immunotoxicity for the PFAS studied so far and the thousands of persistent PFAS lacking data, the most health-protective approach is to manage PFAS as a class and phase out all non-essential uses [1, 85, 86].

Finally, given the clear immunotoxic effects of most PFAS studied to date, the clinical recommendations for PFAS-exposed individuals and communities should be strengthened to consider immune toxicity data for risk management. By knowing the status of their patients’ immune system functions, physicians can make more informed recommendations for behavioral modifications that can protect their patients, particularly in circumstances where risk of infectious disease exposures (e.g., a global pandemic) is high. Physicians are already familiar with recommendations for elderly patients and those taking immunosuppressive medications, and physicians could make similar recommendations for PFAS-exposed patients. Furthermore, the Centers for Disease Control and Prevention (CDC), state health and environmental departments, regulatory agencies, and academic researchers working in communities should inform physicians when they have data indicating that communities have been impacted by PFAS exposure, and share updated clinical guidance, so that the onus is not on individuals to advocate for appropriate treatment.

## Conclusion

A properly functioning immune system is important not just for an individual’s health, but also the health of the population. Immunotoxic chemicals like PFAS pose a threat to both. Discounting the current evidence on immunosuppressive effects of PFAS, particularly with regard to vaccine effectiveness, is not a risk worth taking. Vaccines are now used in many situations, including for childhood illnesses, viruses such as influenza and SARS-CoV-2, and to meet certain work and travel requirements. Yet there is surprisingly little research on their effectiveness in the context of environmental chemical exposures.

In light of the widespread exposure to PFAS and their continued use and production, it is important that the known harmful effects be adequately acknowledged and addressed in chemical regulatory and clinical decision-making contexts. We recommend several changes to current regulatory frameworks to account for immune system effects, particularly the inclusion of biochemical measures as indicators of disease. We also recommend that related clinical guidance be revisited and strengthened to protect individuals, communities, and the public.

### Supplementary Information

Below is the link to the electronic supplementary material.

Supplementary file1 (XLSX 60 KB)
